# The History of Cystinosis: Lessons for Clinical Management

**DOI:** 10.4061/2011/929456

**Published:** 2011-10-13

**Authors:** Paul Goodyer

**Affiliations:** ^1^Department of Pediatrics, McGill University, Montreal, QC, Canada H3H 1P3; ^2^Montreal Children's Hospital, 2300 Tupper Street, Montreal, QC, Canada H3H 1P3

## Abstract

Cystinosis is a rare disorder, and, accordingly, progress on the understanding and treatment of this disease has been relatively slow. Although cystinosis was identified over 100 years ago, the history of cystinosis is marked by a few sudden leaps forward in our understanding rather than by a sustained research effort fuelled by the larger research community. Major conceptual break-throughs include (a) its discovery in 1903, (b) recognition of the renal Fanconi syndrome, (c) realization that tissue accumulation of cystine reflects a defective channel in the lysosomal membrane, (d) translation of this discovery to trials of cysteamine, (e) discovery of the *CTNS* gene, and (f) report of successful stem cell therapy in the cystinotic mouse. This paper focuses on the importance management lessons from these milestones and the potential new therapeutic strategies which may be looming in the near future.

## 1. Cystine Accumulation in Cystinosis

**Figure 1:**
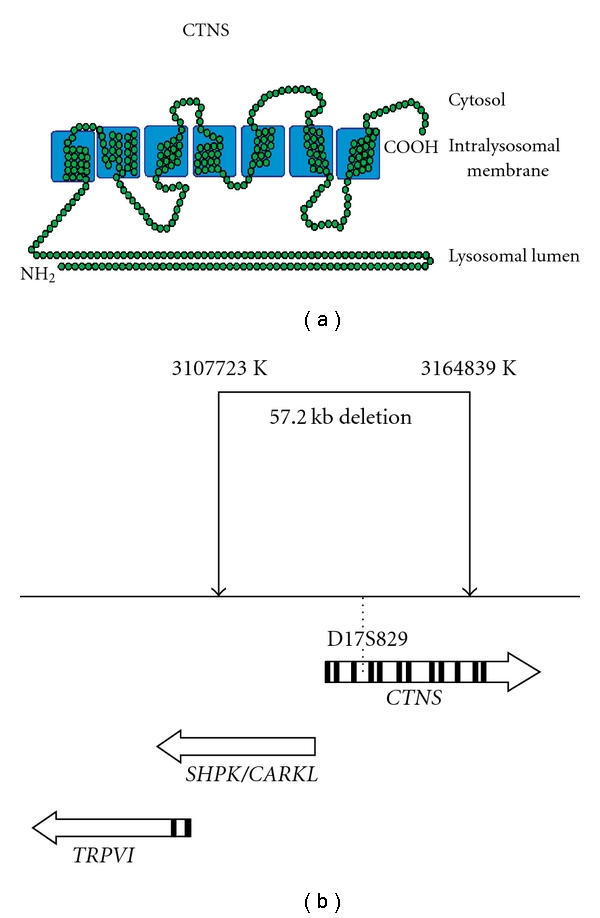
(a) Depiction of *CTNS* protein spanning the lysosomal membrane, (b) the common 57.2 kb deletion extending upstream from *CTNS* exon 10 through the first two exons of *TRPV1*.

Cystinosis as an autosomal recessive disorder caused by mutations of the *CTNS* gene on chromosome 17, which encodes a ubiquitous cystine-specific transporter (cystinosin) in the lysosomal membrane (Figure 1). Since cystinosin facilitates efflux from the lysosome, homozygous *CTNS* mutations result in massive intralysosomal accumulation of cystine in crystals which apparently disrupt the organelle, leading to apoptotic cell death and progressive organ dysfunction. Over 90 mutations of the *CTNS* coding and splice regions have been reported. About half of the cystinosis alleles in the Western populations are caused by a 57.2 kb deletion which extends from the tenth *CTNS* exon through the adjacent SHPK and first two exons of the *TRPV1* (capsaicin receptor) gene ( ). This deletion is thought to have arisen in Northern Germany in about 500 AD. 

An unsavoury character from Zurich, Emil Aberhalden's scientific career was described by Otto Westphal as “a fraud from beginning to end.” Nevertheless, Aberhalden seems to have been the first to identify cystinosis. In 1903, he described a child with severe growth failure in infancy who had cystine crystals in the liver and spleen at autopsy [1]. Tissue accumulation of cystine remains the diagnostic hallmark of cystinosis. Elevation of cystine can be quantified directly from chorionic villus samples at week 9-10 of gestation or can be measured in fetal cells from amniotic fluid prior to 20 weeks of gestation (Jackson Prenatal Dx 2005). At birth, placental cystine (2–5 umoles/g protein) is well above normal (0.1–0.2 umoles/g protein). Although corneal crystals may be seen with a slit lamp by about 1 year of age, these deposits represent coalescence of extracellular cystine left by apoptotic cells and may not be present at birth. As infants develop growth failure at 4–6 months of age, diagnostic elevation of cystine levels can be measured in circulating leukocytes. However, the absolute level of leukocyte cystine varies appreciably from laboratory to laboratory, and lymphocyte cystine (5X normal) is lower than that in polymorphonuclear leukocytes. Since results are expressed as micromoles of cystine per mg cellular protein in the sample, variability in the reported leukocyte cystine level depends, in large part, on how the white cells are isolated at the point of care, rather than from variation induced by the method of cystine measurement (cystine binding assay, automated amino acid analyzer, or HPLC). To minimize variation, the University of California at San Diego has published a protocol for leukocyte isolation (http://biochemgen.ucsd.edu/cystinosis/). This protocol emphasizes prompt leukocyte separation by mixing blood with an equal volume of acid-citrate-dextran solution, allowing the red blood cells to settle by gravity for one hour. Red cells remaining in the upper layer are then lysed; the sample is acidified and frozen for shipping to a central laboratory for protein and cystine determinations. Using this method, leukocyte cystine is nearly undetectable in normal subjects (0–0.12 umoles (half-cystine/gram cell protein) whereas heterozygotes have about 4 times this value, and cystinotic levels are about fifty times normal, usually ranging from 1–10 umoles half-cystine/g protein.

## 2. Cystinosis and the Renal Fanconi's Syndrome

In 1924, Lignac expanded on the observations of Aberhalden to point out that children with cystinosis often present with profound rickets [2]. In 1931, Fanconi was the first to perceive that cystinosis is associated with urinary wasting of substances that are normally reabsorbed during their passage through the renal tubules [3]. The full picture of this tubulopathy was expanded by DeToni, who explained the rickets by documenting urinary phosphate wasting [4] and Debre et al. who noted excretion of organic acids [5]. Fanconi's further contribution to the subject came in 1936, when he recognized the similarities between these cases, referred to the disease as nephrotic-glycosuric dwarfism with hypophosphatemic rickets and suggested that the organic acids found in the urine might be amino acids [6]. In 1947, Dent characterized the urinary amino acid and protein profile of cystinotic children and proved that this was due to defective absorption [7]. 

**Figure 2:**
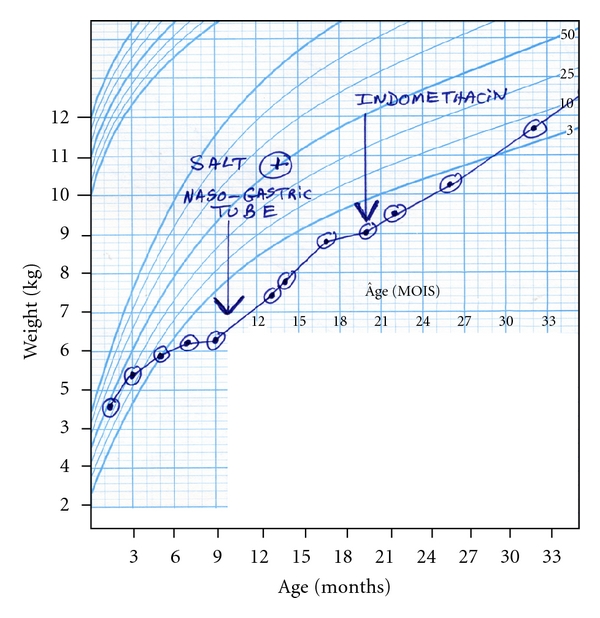
Growth curve for a cystinotic child. (1) Growth velocity fall off at 4–6 months of age improves with salt supplements and increased fluid volume delivered by a nasogastric tube. (2) Slowing of growth velocity at 17–19 months of age is improved by introduction of indomethacin to reduce electrolyte losses by 25–30%.

Unlike most other tissues which deal with the ubiquitous turnover of cellular proteins, the renal proximal tubule must also contend with the enormous daily load of low-molecular-weight proteins contained in glomerular filtrate. These proteins undergo endocytotic uptake as they pass through the proximal tubular lumen and are delivered to lysosomes where they are degraded into their constituent amino acids. Interestingly, newborns may appear normal at birth and exhibit only mild amino aciduria. However, between 4 and 6 months of age proximal tubular dysfunction emerges, and infants fall off their growth curves (Figure 2). By a year of age, apoptotic cell death causes progressive atrophy of the proximal tubule, leading to the “swan neck” deformity described by Clay et al. [8].

With the recognition of the renal Fanconi's syndrome, clinicians were able to extend the lives of cystinotic children by replacing the crucial constituents lost in urine. These therapeutic strategies have been developed over many years and have been reviewed by others [9], but several points are worthy of note as follows.

1. Hypophosphatemic rickets can be largely reversed by divided daily oral supplements of sodium phosphate. However, since phosphate salts are cathartic, the dose should be gradually increased to 50–100 mg/kg/day phosphate over several weeks so that peak serum phosphate levels (at 1 hour) are eventually brought into the low-normal range. Furthermore, since oral phosphate acts as a calcium binder and stimulates parathyroid hormone release, the final oral phosphate dose should be matched by enough calcitriol (10–50 ug/kg/day) to ensure adequate uptake of dietary calcium to maintain normal levels of intact parathyroid hormone.
2. Repair of chronic volume contraction is important for growth. In small infants, this nearly always requires placement of a nasogastric tube and, eventually, a gastrostomy tube. Estimates of urine volume and natriuresis in timed urine collections can then be used to guide fluid intake and salt supplementation and normalize plasma renin level.
3. In small infants, indomethacin (1.5–3 mg/kg/day) may reduce urine volume and electrolyte losses by about one-third. However, it should be accompanied by omeprazole or another modern proton pump inhibitor. Emma has suggested that it may be prudent to discontinue indomethacin as the child approaches school age and has reported that introduction of an ACE inhibitor at this time may prolong renal function [10].
4. Proximal renal tubular acidosis and urinary potassium losses may require large oral supplements of bicarbonate and potassium that may cause vomiting. Correction is best achieved by a mix of sodium bicarbonate, potassium citrate, and potassium chloride.

Although the early years are dominated by the impact of the renal Fanconi's syndrome, untreated children develop a number of nonrenal manifestations. Hair color is usually blond or much lighter than that in the parents. However, it is unclear whether this represents a genetically linked trait or a disturbance of pigmentation, since many examples of black hair have been reported. Corneal crystal deposition becomes increasingly evident during the second year of life. While some children need little therapy, photophobia is common, and most patients begin to use sunglasses in early childhood. Since tear production may be diminished, lubricant eye drops are helpful. If blepharospasm begins to interfere with daily life, children should be treated with topical cysteamine eye drops. Although corneal crystals do not seem to disturb visual acuity, severe untreated corneal involvement may lead to band keratophy which can obscure vision. Progressive dysfunction of the thyroid gland may become evident within the first decade, and elevation of serum TSH levels should prompt oral thyroxine replacement.

## 3. Cystinosis and Progressive Renal Failure

In 1952, Bickel and Smellie pointed out that cystinotic children develop inexorable loss of glomerular filtration despite treatment of the renal Fanconi's syndrome [11]. In untreated cystinotics, multinucleated podocytes are seen in glomeruli [12, 13], and tubular proteinuria is overshadowed by progressive focal segmental glomerulosclerosis associated with nephrotic-range proteinuria and progressive renal insufficiency [14]. By 1974, this prognosis had not changed much, leading Royer to remark in his textbook of Pediatric Nephrology that children with cystinosis usually survive until the age of about 10–12 years, when they succumb to end-stage renal failure [15].

It was about this time, however, that advances in pediatric renal transplantation allowed the first cystinotic children to leap past this roadblock. In 1970, Mahoney et al. reported excellent renal allograft survival in four cystinotic children and pointed out that, as expected, there was no evidence of recurrent cystinosis in the allograft [16]. As cystinotics develop end-stage renal disease, they may have heavy proteinuria and high urine volumes. However, the incidence of graft thrombosis is not different than that in the general population, and children are accustomed to drinking large volumes prior to transplantation [14]. Thus, it is not entirely evident that they need pretransplant nephrectomise, and this decision should be approached carefully.

## 4. Cystinosis and Cysteamine

**Figure 3:**
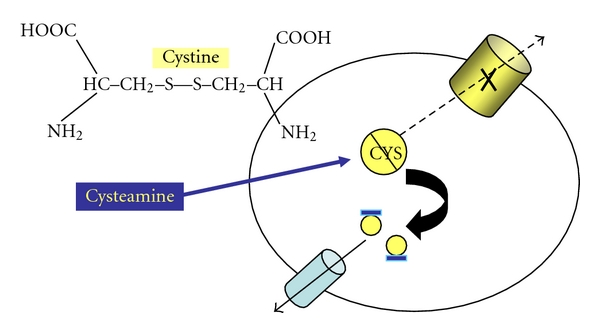
Cystine-depleting effect of cysteamine. Cystine forms mixed sulfides with intralysosomal cystine that are able to efflux into the cytoplasm via alternative lysosomal channels.

In the late 1960s, investigators at the US National Institutes of Health discovered that the accumulation of cystine in patient tissues was due to a defect in efflux of free cystine from lysosomes isolated from cystinotic fibroblasts or leukocytes [17–22]. Patrick and Lake demonstrated ascertained by electron microscopy that the intracellular crystals in cystinotic tissues were surrounded by lysosomal membranes [23]. Reasoning that progressive tissue injury might be ameliorated if cystine could be mobilized from the lysosomes; Schneider reported in 1976 that the sulfhydryl reagent, cysteamine, could react with intralysosomal cystine, permitting efflux of resulting mixed disulfides via alternative channels in the lysosomal membrane (Figure 3) [24]. Gahl went on to show that protracted oral therapy with cysteamine-depleted organ cystine [25] and organized a North American trial of cysteamine that demonstrated slowing of progressive renal failure, particularly in those children who began therapy before the age of two years and were adherent to the prescribed dose (1.3 gm/m^2^, divided qid) [26]. In a retrospective review of 100 adults with cystinosis, Gahl reported that late complications of cystinosis (diabetes mellitus, muscle wasting, hypothyroidism, pulmonary dysfunction, and death) decreased with time on cysteamine therapy [27]. 

**Figure 4:**
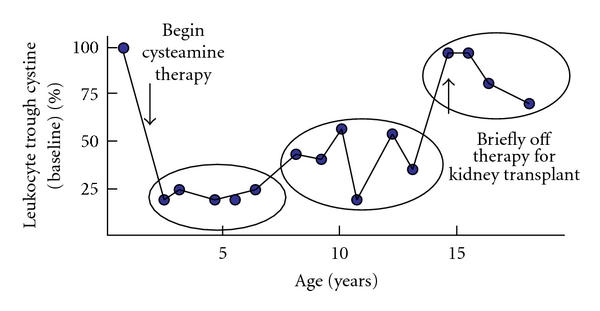
Leukocyte monitoring of leukocyte cystine levels in 50 Canadian children with nephropathic cystinosis. Introduction of oral cysteamine therapy (1.3 g/m^2^/day in four divided doses) in early childhood achieves a rapid reduction of trough 6–8 hours after a single dose. Following a variable “honeymoon period,” mean leukocyte cystine gradually drifts upwards as compliance decreases. Cystine levels return to baseline when stopped briefly for transplant or when teenagers discontinue therapy.

Despite the apparent benefits of long-term cysteamine therapy, there is no question that strict adherence to the medication is difficult. For that reason, leukocyte cystine should be monitored on a regular basis. In early childhood, when most patients receive cysteamine via nasogastric tubes or under parental supervision, leukocyte cystine falls abruptly to about 15% of baseline values which is close to the heterozygous range and usually below 0.5 umoles half-cystine/g protein (Figure 4). However, following a variable “honeymoon period,” mean leukocyte cystine tends to drift gradually upwards, and the number of frankly non-compliant patients increases significantly in adolescence (Goodyer, unpublished). In 2010, Dohil et al. reported their experience comparing enteric-coated cysteamine (25 mg/kg) with the standard cysteamine bitartrate (47 mg/kg) [28]. They demonstrated equivalent mean serum cysteamine levels and noted no significant clinical deterioration while taking the enteric-coated preparation twice a day for 1-2 years [28]. A phase II clinical trial of enteric-coated cysteamine (Raptor Pharmaceutical) is now nearing completion, and it is hoped that adherence will be improved by the twice-a-day dosing schedule.

## 5. Adult Complications of Cystinosis

**Figure 5:**
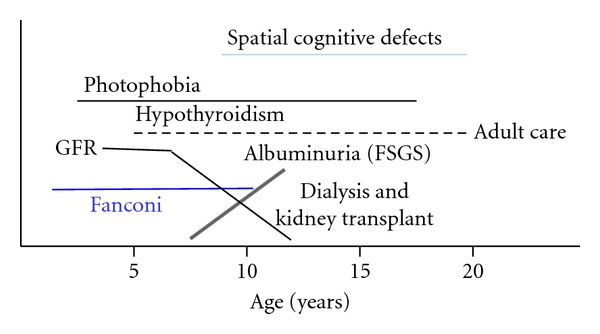
Natural history of untreated cystinosis.

With the advent of renal transplantation and cysteamine therapy, the natural history of cystinosis has been shifted (Figure 5). Nesterova and Gahl have reviewed the late complications of cystinosis, including myopathy, encephalopathy, diabetes mellitus, male infertility, retinal degeneration, hypothyroidism and coronary artery calcifications [29]. In general, the severity of these complications correlates with the number of years without cysteamine treatment. However, cysteamine therapy does not prevent the need for eventual renal transplantation and cannot eliminate the many debilitating complications that arise in adulthood. In 1988, Sonies et al. reported the case of a 20-year-old male with distal muscle atrophy and progressive loss of the ability to sit without support, but sensation and nerve conduction were intact. In a review of 101 posttransplant patients, about half had evidence of significant myopathy, particularly debilitating was the loss of pharyngeal musculature causing dysphagia and oromotor dysfunction interfering with speech [30]. Myopathy contributes to light exercise intolerance and pulmonary dysfunction as well.

Trauner et al. has recently described the subtle spatial perception deficit in children with cystinosis [31]. Young cystinotic children might have subtle learning disabilities that could be overcome with learning interventions that diminish reliance on spatial perception skills. After the age of twenty, untreated cystinotic adults may develop cortical dysfunction with progressive confusion, tremor, and seizures [32]. Broyer et al. reported that only 5% of patients have evidence of encephalopathy at age 23, but this increases rapidly to 45% by age 27 [33]. Interestingly, Broyer et al. found that late introduction of cysteamine seemed to improve some manifestations of the encephalopathy [33]. Mueller described a cystinotic woman who developed some memory loss with mild cortical atrophy at age of 29 evident by magnetic resonance imaging. With introduction of cysteamine, cerebral function and cortical atrophy were stable, but distal myopathy continued to progress. If this case is representative, it may be difficult to avert muscle wasting in adult cystinosis. Although growth hormone has been used to accelerate somatic growth in cystinotic children, its effects on muscle wasting in the adult have not been studied. 

Experience with cystinosis in adulthood offers several management lessons. Firstly, that cysteamine therapy should be started as early as possible and continued throughout life; renal transplantation does not diminish the need for continued therapy, and even late introduction of cysteamine may provide clinical benefits. Secondly, adherence to cysteamine therapy is especially difficult during teenage years and strategies to improve compliance and facilitate the transition to adult care systems are crucial. Thirdly, the many forms of organ dysfunction that arise during adulthood warrant specialized cystinosis-specific outpatient care units, since management usually requires expertise well beyond the skills of the nephrologist who may preside over care at the time renal transplantation is needed.

## 6. Cystinosis and Stem Cell Therapy

In 2009, Syres et al. reported successful hematopoietic stem cell therapy of cystinosis in mice [34]. Cells from a wild-type mouse infused into *CTNS *(−/−) recipients were able to home to damaged tissues, reducing tissue cystine levels by 90% and normalizing organ function. This remarkable observation raises this immediate question of whether a similar strategy might be feasible in humans. One scenario would involve standard bone marrow transplantation, in which bone marrow stem cells from a healthy donor are infused into the cystinotic patient who has undergone marrow-ablative chemotherapy. Alternatively, the patient's own stem cells might be corrected by transfection with the wild-type *CTNS* gene *in vitro *and reinfused. Although there remain a number of practical problems to solve before human trials are considered, work in the mutant mouse model has opened a new hope for the treatment of cystinosis.
